# The Impact of Lipid Handling and Phase Distribution on the Acoustic Behavior of Microbubbles

**DOI:** 10.3390/pharmaceutics13010119

**Published:** 2021-01-19

**Authors:** Simone A.G. Langeveld, Inés Beekers, Gonzalo Collado-Lara, Antonius F. W. van der Steen, Nico de Jong, Klazina Kooiman

**Affiliations:** 1 Thorax Center, Biomedical Engineering, Erasmus University Medical Center, 3000 CA Rotterdam, The Netherlands; inesbeekers@gmail.com (I.B.); g.colladolara@erasmusmc.nl (G.C.-L.); a.vandersteen@erasmusmc.nl (A.F.W.v.d.S.); n.dejong@erasmusmc.nl (N.d.J.); k.kooiman@erasmusmc.nl (K.K.); 2 Acoustical Wavefield Imaging, Delft University of Technology, 2628 CJ Delft, The Netherlands

**Keywords:** ultrasound contrast agents, phospholipid coating, ligand distribution, cholesterol, acoustic response, microbubble, lipid phase

## Abstract

Phospholipid-coated microbubbles are ultrasound contrast agents that can be employed for ultrasound molecular imaging and drug delivery. For safe and effective implementation, microbubbles must respond uniformly and predictably to ultrasound. Therefore, we investigated how lipid handling and phase distribution affected the variability in the acoustic behavior of microbubbles. Cholesterol was used to modify the lateral molecular packing of 1,2-distearoyl-*sn*-glycero-3-phosphocholine (DSPC)-based microbubbles. To assess the effect of lipid handling, microbubbles were produced by a direct method, i.e., lipids directly dispersed in an aqueous medium or indirect method, i.e., lipids first dissolved in an organic solvent. The lipid phase and ligand distribution in the microbubble coating were investigated using confocal microscopy, and the acoustic response was recorded with the Brandaris 128 ultra-high-speed camera. In microbubbles with 12 mol% cholesterol, the lipids were miscible and all in the same phase, which resulted in more buckle formation, lower shell elasticity and higher shell viscosity. Indirect DSPC microbubbles had a more uniform response to ultrasound than direct DSPC and indirect DSPC-cholesterol microbubbles. The difference in lipid handling between direct and indirect DSPC microbubbles significantly affected the acoustic behavior. Indirect DSPC microbubbles are the most promising candidate for ultrasound molecular imaging and drug delivery applications.

## 1. Introduction

Microbubbles are small gas bubbles (diameter 1–10 µm) that are clinically used as ultrasound contrast agents for non-invasive diagnostic imaging of blood perfusion [1]. Targeted microbubbles are employed for molecular imaging of inflammation, tumors, and cardiovascular disease [2]. Other types of microbubbles are being developed specifically for drug delivery [3]. All of these applications make use of the compression and expansion of the microbubble gas core upon ultrasound insonification. These microbubble vibrations produce a nonlinear response, including super- and subharmonic oscillations, which can be differentiated from the surrounding tissue to form a contrast-enhanced image [1]. Additionally, this acoustic response can induce bioeffects on nearby cells–resulting in enhanced uptake or extravasation of drug molecules [4]. Successful translation to the clinical use of microbubbles for molecular imaging and enhanced drug delivery is currently challenged, however, by the microbubbles’ unpredictable acoustic behavior.

To stabilize the gas core, microbubbles are usually coated with a phospholipid monolayer, proteins, or polymers. For a schematic representation, the reader is referred to recent reviews on microbubbles [5,6]. The coating reduces surface tension and gas diffusion [7]. If phospholipids or polymers are used as microbubble coating, a ligand can be attached for molecular imaging [8], and they can be loaded with a drug for localized delivery [3]. The physicochemical properties of the microbubble coating, such as the shell elasticity and viscosity, are related to the acoustical properties, such as the resonance frequency and the damping coefficient [9,10]. Therefore, the composition of the microbubble coating can affect the acoustical properties. For instance, the use of a phospholipid molecule with a longer acyl chain length, 1,2-distearoyl-*sn*-glycero-3-phosphocholine (DSPC; C18), resulted in a higher shell elasticity and more acoustic stability than the use of a shorter acyl chain length phospholipid, 1,2-dipalmitoyl-*sn*-glycero-3-phosphocholine (DPPC; C16) [11]. Besides the shell elasticity, acyl chain length has also been shown to affect the half-life of microbubbles, with longer acyl chain length resulting in more stable size distribution and ultrasound signal over time [12].

Since microbubbles are generally coated with a mixture of phospholipids and a PEGylated emulsifier, the physicochemical properties are determined by the miscibility and lipid phase behavior. Molecules in the microbubble coating can be in the liquid expanded (LE) or liquid condensed (LC) phase, resulting in distinctive microstructures. These microstructures can be altered by using different types of phospholipids [13], changing the ratio between phospholipid and emulsifier, or heating and cooling of the microbubble coating [14]. Microstructures formed by lipid phase separation have been shown to affect the subharmonic response to ultrasound [15]. The effect of lipid phase separation on the subharmonic response to ultrasound has been characterized previously in three types of microbubbles with different levels of lipid phase separation: 20%, 50% or 80% of the microbubbles had LC phase domains. Each microbubble type had a peak subharmonic response at a different microbubble size, suggesting that microstructures in the coating affect the acoustical properties of a microbubble [15]. The microbubble coating can also be altered by the distribution of the phospholipid and PEGylated-emulsifier molecules over the microbubble coating, depending on the lipid handling prior to microbubble production by probe sonication. The use of organic solvent resulted in a more homogeneous ligand distribution than the use of aqueous solutions only [16]. The effect of lipid handling on the acoustic response of microbubbles, however, has not been investigated.

For both ultrasound molecular imaging and drug delivery, it is important that all microbubbles respond uniformly and predictably to ultrasound. Currently available microbubbles respond to ultrasound in a heterogeneous way [11,17], even when they are the same size [18]. While it is thought this variability in response could be due to the microstructures in the microbubble coating, this is challenging to confirm because it can only be investigated by looking at single microbubbles. Different approaches have been used to record a single microbubble’s response to ultrasound, including an ultra-high-speed camera to image the microbubble during insonification [19], recording the acoustic response [15] or optical scattering [20], and photo-acoustic techniques [21]. Until recently, however, no techniques were available to image both the lipid phase distribution in 3D and the acoustic response of the same microbubble. In this regard, the challenge lies in the time scale (µs) and optical resolution (µm) needed to record the lipid phase distribution and response to ultrasound of a single microbubble.

The purpose of this study was to relate the effects of lipid handling and phase distribution before microbubble production to the acoustic behavior of phospholipid-coated microbubbles. Cholesterol can modify the lateral molecular packing of phospholipids in a monolayer, resulting in a single liquid phase [22,23,24]. While microbubbles with cholesterol in their coating have been produced before [25,26], the effect of cholesterol on the lipid phase separation in microbubbles has not been studied. To determine this effect in the microbubble coating, we made microbubbles by probe sonication with DSPC as the main lipid and varying concentrations of cholesterol. The lipid phase distribution and ligand distribution in the microbubble coating were imaged using high-axial-resolution 4Pi confocal microscopy. To assess the acoustic response and variability in the acoustic behavior, we used a unique system combining a confocal microscope with the Brandaris 128 ultra-high-speed camera. With this system, the lipid phase separation (in nanometer resolution) and acoustic response to ultrasound (in nanosecond resolution) were captured at a single microbubble level.

## 2. Materials and Methods

### 2.1. Materials

DSPC was provided by Lipoid GmbH (Ludwigshafen, Germany). PEG40-stearate and cholesterol were purchased from Sigma-Aldrich (Zwijndrecht, The Netherlands), 1,2-distearoyl-*sn*-glycero-3-phosphoethanolamine-*N*-carboxy-(polyethylene glycol) (DSPE-PEG2000) was purchased from Iris Biotech GmbH (Marktredwitz, Germany), and 1,2-distearoyl-*sn*-glycero-3-phosphoethanolamine-*N*-biotinyl(polyethylene glycol) (DSPE-PEG2000-biotin) was purchased from Avanti Polar Lipids (Alabaster, AL, USA). Perfluoro butane (C_4_F_10_) was purchased from F2 Chemicals (Preston, UK), and argon gas was purchased from Linde Gas Benelux (Schiedam, the Netherlands). Streptavidin Oregon Green 488 was purchased from BioSynthesis (Louisville, TX, USA), and Lissamine rhodamine B 1,2-dihexadecanoyl-*sn*-glycero-3-phosphoethanolamine, triethylammonium salt (rhodamine-DHPE) was purchased from Thermo Fisher (Waltham, MA, USA).

### 2.2. Microbubble Production

Biotinylated lipid-coated microbubbles with a C_4_F_10_ gas core were made as described previously [27], by probe sonication at 20 kHz with a Sonicator ultrasonic processor XL2020 at a power setting 10 (HeatSystems, Farmingdale, NY, USA) for 10 s. Three types of microbubbles were made by altering the production method or adding cholesterol to the microbubble coating. For microbubbles without cholesterol, the coating components (84.8 mol% DSPC; 8.2 mol% PEG40-stearate; 5.9 mol% DSPE-PEG2000; 1.1 mol% DSPE-PEG2000-biotin) were prepared with either an indirect or a direct method as described previously [16]. In short, for the indirect method, the components were dissolved in chloroform/methanol (9:1 vol/vol), the solvent was evaporated using argon gas, and the obtained lipid film was dried overnight under vacuum. The lipid film was then dispersed in saline solution (0.9% NaCl, saturated with C_4_F_10_) with a final concentration of 2.5 mg/mL DSPC, 0.625 mg/mL PEG40-stearate, 0.625 mg/mL DSPE-PEG2000 and 0.125 mg/mL DSPE-PEG2000-biotin. The fluorescent dye rhodamine-DHPE (0.01 mol%) was added to image the lipid phase separation in the microbubble coating. The solution was placed in a sonicator bath for 10 min, and the probe sonicator was used at power setting 3 for 5 min. For the direct method, the coating components (84.8 mol% DSPC; 8.2 mol% PEG40-stearate; 5.9 mol% DSPE-PEG2000; 1.1 mol% DSPE-PEG2000-biotin) were dispersed directly in C_4_F_10_-saturated saline solution with a final concentration of 2.5 mg/mL DSPC, 0.625 mg/mL PEG40-stearate, 0.625 mg/mL DSPE-PEG2000 and 0.125 mg/mL DSPE-PEG2000-biotin. Fluorescent dye rhodamine-DHPE (0.01 mol%) was added before sonication.

Microbubbles with cholesterol, referred to as DSPC-cholesterol microbubbles, were produced with the indirect method only since cholesterol is insoluble in an aqueous medium, and the organic solvent was required to mix all microbubble coating components [28]. Cholesterol was added (7, 10, 12, 14, or 32 mol%) to the ternary mixture of coating components: DSPC, PEG40-stearate, DSPE-PEG2000, and DSPE-PEG2000-biotin (molar ratio 84.8/8.2/5.9/1.1) in chloroform/methanol (9:1 vol/vol). The lipids were then dried to form a lipid film and dispersed in saline solution, as described above, with 0.02 mol% rhodamine-DHPE added for fluorescent labeling of the microbubbles. All types of microbubbles were produced by sonicating under a constant flow of C_4_F_10_.

### 2.3. Physicochemical Characterization

To image the ligand distribution, fluorescent ligand streptavidin Oregon Green 488 was conjugated to the biotinylated microbubbles as described previously [29]. Briefly, microbubbles were first washed by flotation: 0.9 mL microbubble suspension was placed in a 3 mL syringe and topped with 2.1 mL saline solution saturated with C_4_F_10_. After 45 min, the subnatant was drained, and the microbubbles were resuspended in 0.3 mL saline solution saturated with C_4_F_10_. Then, 22.5 µL of streptavidin-Oregon Green 488 (2 mg/mL) was allowed to incubate with 0.7–1.0 × 10^8^ microbubbles for 30 min on ice. The excess of streptavidin was washed away by flotation as described above, with resuspension of the microbubbles in 0.2 mL saline solution.

To measure the microbubble size distribution and concentration, a Coulter Counter Multisizer 3 (Beckman Coulter, Mijdrecht, The Netherlands) was used. To quantify particles between 1 and 30 µm, a 50 µm aperture tube was used. To evaluate the polydispersity of the samples, the span value was calculated, defined as (*d*90 − *d*10%)/*d*50%, where *d*90, *d*10 and *d*50% are the microbubble diameters below which 90, 10 and 50% of the cumulative number of microbubbles was found. Samples were measured after the first flotation wash and again after conjugation with streptavidin Oregon Green 488.

The streptavidin-conjugated microbubbles were imaged by microscopy as described by Langeveld et al. [16]. In short, the microbubbles were placed between quartz glass in 87% glycerol (*v*/*v* in phosphate-buffered saline) to reduce Brownian motion and imaged with a Leica TCS 4Pi confocal laser-scanning microscope [30]. An axial resolution up to 90 nm was achieved with a matched pair of aligned opposing 100× glycerol HCX PL APO objective lenses (numerical aperture 1.35). For excitation of Oregon Green 488, a 488 nm laser was used, and for excitation of rhodamine-DHPE, a 561 nm laser was used. Images were recorded in 3D as *y*-stacked *xz*-scans in a green (500−550 nm) and red (580−640 nm) spectral channel. The “voltex” function was used to volume-render the image stacks with AMIRA (Version 2020.2, FEI, Mérignac Cedex, France).

Quantitative analysis was performed on the 4Pi microscopy data using custom-developed image analysis software in MATLAB (Mathworks, Natick, MA, USA), based on the method described by Langeveld et al. [16]. The microbubble coating was subdivided into 32 parts, of which the mean fluorescence pixel intensity (*I*_part_ for the green channel and *I*_part-rhod_ for the red channel) was calculated. The median intensity of all parts (*I*_median_ for the green channel and *I*_median-rhod_ for the red channel) was calculated per microbubble. To evaluate the ligand distribution, parts were classified as inhomogeneous when the absolute difference between *I*_part_ and *I*_median_ was more than two-thirds times the value of *I*_median_ (i.e., |*I*_part_ − *I*_median_| > 2/3 × *I*_median_), and the percentage of inhomogeneous parts was calculated per microbubble. To evaluate the lipid phase distribution, parts were classified as LC phase when the value of *I*_part-rhod_ was less than one-third of *I*_median-rhod_ (i.e., *I*_part__-rhod_ < 1/3 × *I*_median__-rhod_). The LC phase surface area was first calculated in µm^2,^ and then a percentage of the total analyzed surface area per microbubble. Before evaluating the ligand distribution or the lipid phase distribution, an additional normalization step was included in the image analysis. This step corrected for a difference in fluorescence intensity between the center and the top or bottom of the microbubbles, likely caused by attenuation of the laser light leading to a lower fluorescence signal at the center of the sample. The normalization factor was calculated based on the median *I*_part_ (for the green channel) or the median *I*_part-rhod_ (for the red channel) per angular part from all microbubbles (Supplemental Figure S1). To determine the number of microbubbles with buckles, the microbubble coating was manually scored for fluorescent signal outside and attached to the microbubble coating, based on the red channel (rhodamine-DHPE signal). Only bright spots with 1 µm diameter or larger were classified as a buckle.

### 2.4. Acoustical Characterization

To study both the acoustical behavior and the lipid phase separation of single microbubbles simultaneously, the combined confocal microscopy and Brandaris 128 ultra-high-speed camera system was used [31]. Microbubble spectroscopy was employed to characterize the acoustic behavior of single microbubbles as described previously [11,32]. Microbubbles were washed by flotation once and counted using the Coulter Counter Multisizer 3, as described above. An acoustically compatible [32] CLINIcell (MABIO, Tourcoing, France) with 50 µm membranes (25 µm^2^) was first blocked with 12 mL of 2% (*w/v*) bovine serum albumin (BSA) in phosphate-buffered saline (PBS) for 1 h, to avoid unspecific microbubble binding to the membranes. The CLINIcell was washed three times with PBS before inserting 12 mL of 10^5^ microbubbles/mL in PBS. Next, the CLINIcell was placed underwater in the experimental setup and kept at room temperature for up to 2 h. To study the lipid phase separation, the custom-built confocal microscope (Nikon Instruments, Amsterdam, The Netherlands) was used with a 561 nm laser to excite rhodamine-DHPE and emitted light was detected in a 595/50 nm channel. *Z*-stacks with 0.4 µm steps were acquired with a CFI Plan 100 × W objective of single microbubbles directly before and after insonification. To perform microbubble spectroscopy, each individual microbubble was insonified over a range of transmitting frequencies (*f_T_*) from 1 to 4 MHz in steps of 200 kHz. The microbubbles were insonified with 8-cycle Gaussian tapered sine wave bursts either at 50 kPa or first at 20 kPa and then at 150 kPa external peak negative pressure (PNP), generated by a Tabor 8026 arbitrary waveform generator (AWG, Tabor Electronics, Tel Hanan, Israel). The signal was first attenuated by a 20-dB attenuator (Mini-Circuits, Brooklyn, New York, NY, USA), then amplified by a broadband amplifier (ENI A-500, Electronics and Innovation, Rochester, New York, NY, USA), and finally transmitted to the microbubble sample at a 45° incidence angle with a single-element transducer (1–9 MHz bandwidth, 25 mm focal distance, −6 dB beamwidth at 1 MHz of 1.3 mm, PA275, Precision Acoustics, Dorchester, UK), which was calibrated using a 1-mm needle hydrophone (Precision Acoustics, Dorchester, UK) in water. The Brandaris 128 ultra-high-speed camera [33], coupled with the confocal microscope [31], was used to record the microbubble oscillation behavior at approximately 17 million frames/s. First, a recording was made without ultrasound to establish the initial microbubble size. Next, 16 recordings at 50 kPa PNP, or 16 recordings at 20 kPa PNP and then 16 recordings at 150 kPa PNP were made of a single microbubble upon ultrasound insonification at the different transmit frequencies with 80 ms in between recordings. To avoid any effects from nearby microbubbles on the oscillation behavior, only microbubbles which were at least 0.7 mm from other microbubbles were investigated.

To quantify microbubble oscillation, custom-developed image analysis software in MATLAB was used to determine the change in microbubble radius as a function of time (*R*—*t* curve) [19]. As previously described, the resonance frequency and shell parameters can be obtained from the spectroscopy dataset [11,19]. Briefly, the relative oscillation amplitude (*x*_0_) of each microbubble was defined as the maximum of the filtered *R-t* curve (a third-order Butterworth bandpass filter centered at *f_T_* with a 300 kHz bandwidth) and divided by the resting size of the microbubble (*R*_0_; mean size of the first five frames). Next, for each *f_T_*, the *x*_0_ obtained at 50 kPa were fitted to the harmonic oscillator model:

(1) $$x_{0}=\frac{\left|P\right|/\left(4\pi^{2}\rho R_{0}^{2}\right)\;}{\sqrt{{\left(f_{0}^{2}-f_{T}^{2}\right)}^{2}+{\left(\delta f_{T}f_{0}\right)}^{2}}}$$

with *P* being the acoustic pressure and *ρ* = 10^3^ kg/m^3^ being the density of water. The eigenfrequency (*f*_0_) of the microbubble is defined as:

(2) $$f_{0}=\;\frac{1}{2\pi }\sqrt{\frac{1}{\rho R_{0}^{2}}\left[3\gamma P_{0}+\frac{2\left(3\gamma -1\right)\sigma_{w}}{R_{0}}+\frac{4\chi }{R_{0}}\right]}$$

with *γ* = 1.07, the ratio of specific heats for C_4_F_10_, *P*_0_ = 10^5^ Pa the ambient pressure, *σ_w_* = 0.072 N/m the surface tension in water, and *χ* the microbubble shell elasticity. The damping coefficient (δ) is given by:

(3) $$\delta =\frac{\omega_{0}R_{0}}{c}+2\frac{4\mu }{R_{0}^{2}\rho \omega_{0}}+\frac{4\kappa_{s}}{R_{0}^{3}\rho \omega_{0}}$$

with *ω*_0_ = 2π*f*_0_, *c* = 1500 m/s the speed of sound in water, µ = 10^−3^ Pa·s the viscosity of water and *κ_s_* the microbubble shell viscosity. The resonance frequency is defined by $f_{res}=f_{0}\sqrt{1-\delta^{2}/2}$.

The variability in the acoustical response of each microbubble type was quantified by determining the interquartile range (IQR) of the relative oscillation amplitude (*x*_0_) at each *f*_T_ and in diameter bins of 0.5 µm (*N* > 3 per bin). Since the microbubbles deflated after insonification, the acoustic stability was evaluated by quantifying the relative diameter decrease upon insonification as (*D*_0_ − *D_end_*)/*D*_0_, with *D*_0_ the mean microbubble diameter of all 128 frames of the first recording without ultrasound and *D_end_* the mean microbubble diameter of the last ten frames of the last recording.

The nonlinear behavior of microbubbles was assessed by calculating the fast fourier transforms (FFTs) of the *R*-*t* curves. The noise level of each microbubble was determined by the FFT of the first recording before the ultrasound. A microbubble was categorized as exhibiting nonlinear behavior when in at least two recordings it showed a detectable peak in the FFT (using the *islocalmax* function in MATLAB) around ½·*f_T_* for the subharmonic or around 2·*f_T_* for the second harmonic and the peak’s amplitude was at least 6 dB above the noise level. If so, then the amplitude of the nonlinear component was defined as the maximum FFT amplitude in a 300 kHz bandwidth around ½·*f_T_* for the subharmonic component and around 2·*f_T_* for the second harmonic component and normalized to the fundamental at *f_T_*.

Finally, the confocal microscopy recordings were scored manually for the presence of buckles (none, single, multiple, or extensive) before and after the ultrasound and for change in the microbubble coating before and after ultrasound (unchanged, buckles formed, coating material shed). Only bright spots with 1 µm diameter or larger were classified as the buckle (Supplemental Figure S2). Microbubbles between 4.5 and 6.0 µm in diameter were manually scored for the LC domain size as well (mostly large, large and small, undefined). The relationship between these classifications and the acoustical data were evaluated to determine the effect of the lipid phase distribution and buckling in the microbubble coating on the resulting acoustic response. To rule out size-dependent differences in oscillation amplitude, only microbubbles with an initial diameter in the range of 4.5–6.0 µm were included in this analysis.

### 2.5. Statistics

Statistical analysis was performed using IBM SPSS Statistics 25 for all 4Pi microscopy image analysis. Statistical analysis for the acoustical characterization was performed using MATLAB. A Shapiro–Wilk test was used to assess the distribution of the data. For data that were normally distributed, a regular *t*-test was used to analyze the differences between groups. For all other data, the Mann−Whitney *U* test was used to test the difference between groups. Differences between groups were only tested for *N* > 2. Pearson’s correlation tests were performed to assess the correlation between parameters.

## 3. Results

### 3.1. Physicochemical Characterization

**Figure 1:**
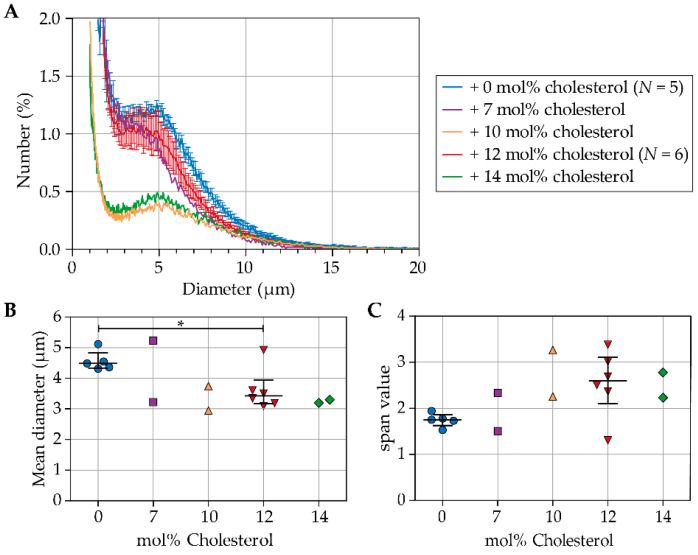
(**A**) Number weighted size distribution, (**B**) number weighted mean diameter (μm), and (**C**) span value of indirect DSPC-based microbubbles with cholesterol in a range from 0 to 14 mol%. In B and C, each symbol represents one batch of microbubbles; jittering was applied to avoid overlapping. The overlaid black lines represent the median and interquartile range. Statistical significance is indicated with * *p* < 0.05.

Figure 1A presents the number weighted size distributions of indirect DSPC-based microbubbles with and without cholesterol. For microbubbles without cholesterol (0 mol%; *N* = 5) and microbubbles with 12 mol% cholesterol (*N* = 6), the size distributions of batches for 4Pi microscopy and for acoustic experiments are both included, and the mean number (%) per diameter is shown with the standard error of the mean (SEM). For microbubbles with 7, 10, and 14 mol% cholesterol a representative curve is shown from 2 batches, as these types of microbubbles were produced for 4Pi microscopy only. The concentration of microbubbles ranged from 2.78 × 10^8^ to 1.17 × 10^9^ microbubbles per mL (Supplemental Table S1). The indirect DSPC-based microbubbles without cholesterol had more particles with diameter >3 µm than all types of microbubbles with cholesterol in the coating. Indirect DSPC-based microbubbles with 32 mol% cholesterol in the coating were highly unstable, with a concentration too low for measurement of the size distribution. Therefore, indirect DSPC-based microbubbles with 32 mol% cholesterol were not investigated further.

Figure 1B shows the mean diameter (μm) of indirect DSPC-based microbubbles without cholesterol and with 7, 10, 12, or 14 mol% cholesterol. Microbubbles with 12 mol% cholesterol had a smaller mean diameter than those without cholesterol (*p* = 0.045). Figure 1C shows the width of the size distributions represented as the span value. The size distributions of microbubbles with 12 mol% cholesterol were more polydisperse than those of microbubbles without cholesterol (*p* = 0.068).

**Figure 2:**
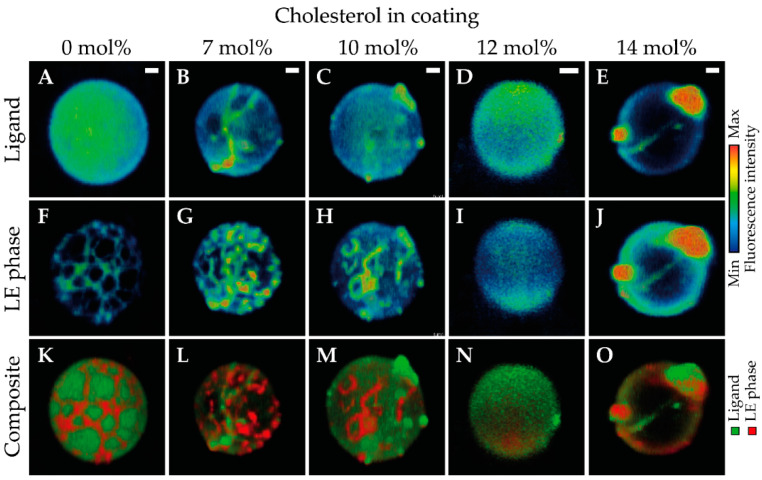
Selected views of 4Pi confocal microscopy *y*-stacks of indirect 1,2-distearoyl-*sn*-glycero-3-phosphocholine (DSPC)-based microbubbles without cholesterol (**A**,**F**,**K**, diameter (*d*) = 6.4 µm, liquid condensed (LC) phase area 35%), with 7 mol% cholesterol (**B**,**G**,**L**, *d* = 5.6 µm, LC phase area 22%), with 10 mol% cholesterol (**C**,**H**,**M**, *d* = 6.1 µm, LC phase area 22%), with 12 mol% cholesterol (**D**,**I**,**N**, *d* = 3.6 µm, LC phase area 7%), and with 14 mol% cholesterol (**E**,**J**,**O**, *d* = 5.8 µm, LC phase area 22%) in the phospholipid coating. Images show the ligand distribution (**A**–**E**; Oregon Green 488), liquid expanded (LE) phase (**F**–**J**; rhodamine-DHPE), and composite view (**K**–**O**). Scale bars are 1 µm.

**Figure 3:**
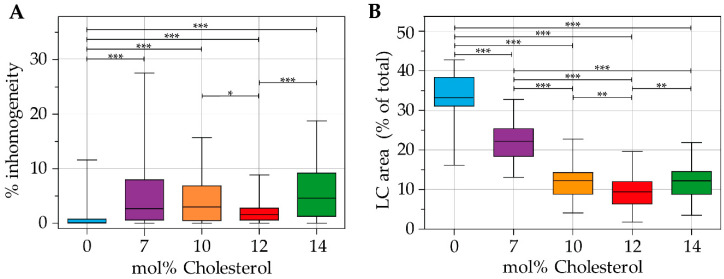
(**A**) Parts classified as inhomogeneity (%) in the ligand distribution, and (**B**) size of the LC area (% of total surface area) of indirect DSPC microbubbles without cholesterol (*N* = 58), with 7 mol% (*N* = 34), 10 mol% (*N* = 40), 12 mol% (*N* = 61), and with 14 mol% (*N* = 45) cholesterol in the coating. Boxplots show the median and interquartile range with whiskers from minimum to maximum. Statistical significance is indicated with * *p* < 0.05, ** *p* < 0.01, or *** *p* < 0.001.

The ligand and lipid phase distribution in the microbubble coating were imaged in indirect DSPC-based microbubbles without cholesterol (*N* = 58), with 7 mol% cholesterol (*N* = 34), with 10 mol% cholesterol (*N* = 40), with 12 mol% cholesterol (*N* = 61), and with 14 mol% cholesterol (*N* = 45). Images were recorded of at least two batches of microbubbles for all formulations, with microbubble diameters ranging from 2.2 µm to 8.7 µm. Typical examples of all formulations are presented in Figure 2. The ligand distribution is shown in the top row, the LE phase in the middle row, and a composite of both channels in the bottom row. Figure 3 shows a quantitative analysis of the 4Pi confocal microscopy images, with the calculated ligand distribution inhomogeneity in Figure 3A and the LC phase relative to the total surface area analyzed per microbubble in Figure 3B. Indirect DSPC-based microbubbles without cholesterol had a homogeneous ligand distribution (Figure 2A, Figure 3A). The inhomogeneity of the ligand distribution can be observed in Figure 2B,C,E, where the ligand is enriched in some areas of the microbubble surface. All indirect DSPC-cholesterol microbubbles had a significantly more heterogeneous ligand distribution compared to those without cholesterol (Figure 2B–E, Figure 3A). Microbubbles with 12 mol% cholesterol had a more homogeneous ligand distribution than those with 7 mol% cholesterol (*p* = 0.070), 10 mol% cholesterol (*p* = 0.040), and 14 mol% cholesterol (*p* < 0.001).

The lipids were phase-separated in indirect DSPC-based microbubbles without cholesterol, as shown in Figure 2F and quantified in Figure 3B. The fluorescent dye rhodamine-DHPE was enriched in bright interdomain regions (i.e., LE phase) and absent in LC domains. In indirect DSPC-cholesterol microbubbles, the LC domains were less pronounced compared to those without cholesterol (Figure 2G–J). With increasing concentrations of cholesterol up to 12 mol%, the lipid phase distribution was increasingly affected, as reflected by quantification of the LC phase area (Figure 3B). Microbubbles without cholesterol had a significantly larger surface area in the LC phase than those with cholesterol in their coating. Microbubbles with 7 mol% cholesterol displayed LE phase areas with an enriched fluorescent dye (Figure 2G) and had a significantly larger surface area in the LC phase than those with more cholesterol in their coating. Microbubbles with 10 mol% cholesterol displayed LE phase areas as well (Figure 2H). Microbubbles with 12 mol% cholesterol had a homogeneous distribution of the fluorescent dye rhodamine-DHPE (Figure 2I), with the smallest LC phase area per microbubble of all formulations (Figure 3B). In microbubbles with 14 mol% cholesterol, rhodamine-DHPE was not only distributed homogeneously in the coating but also present in buckles on the outside of the coating (Figure 2J). The LC phase area in microbubbles with 14 mol% cholesterol was comparable to the LC phase area in microbubbles with 10 mol% cholesterol (Figure 3B).

**Figure 4:**
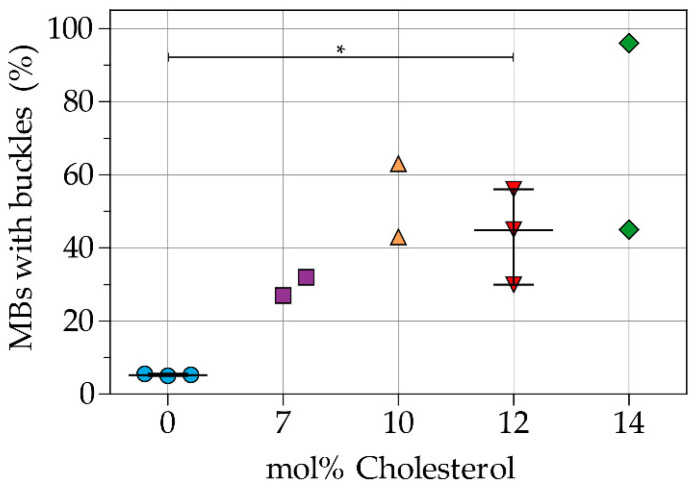
Percentage of microbubbles (MBs) with buckles per batch of indirect DSPC-based microbubbles without cholesterol and with 7, 10, 12, or 14 mol% cholesterol. Each symbol represents one batch of microbubbles. Overlaid black lines represent the median and interquartile range. Statistical significance is indicated with * *p* < 0.05.

Figure 4 shows the percentage of indirect DSPC-based microbubbles with buckles per batch. An example of a microbubble with buckles is shown in Figure 2J,O. Microbubbles without cholesterol in the coating had the lowest incidence of buckles. Microbubbles with 12 mol% cholesterol in the coating had a higher incidence of buckles (*p* = 0.050) than those without cholesterol. Furthermore, the variability between batches increased with higher concentrations of cholesterol.

### 3.2. Acoustical Characterization

**Figure 5:**
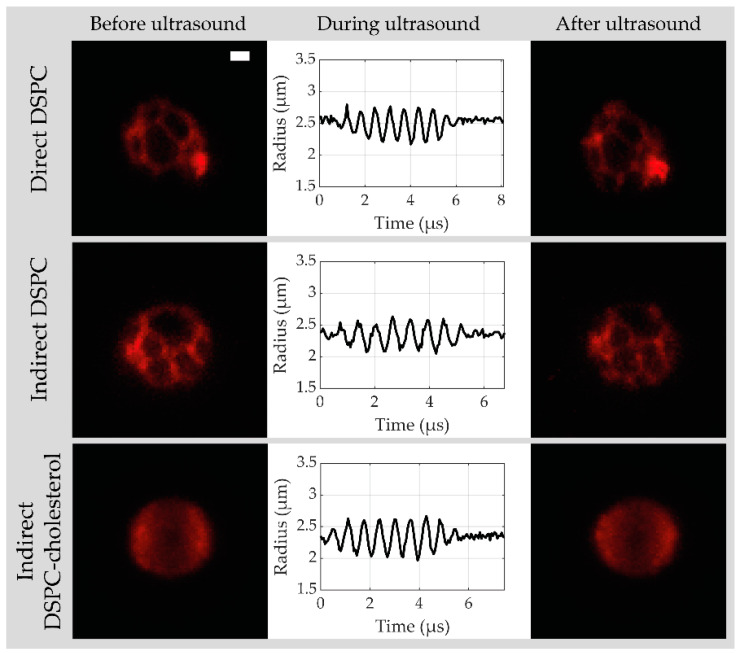
Maximum intensity projections of confocal *z*-stack from direct DSPC, indirect DSPC, and indirect DSPC-cholesterol (12 mol%) microbubbles with the LE phase in red, before and after ultrasound, with the microbubble radius as a function of time obtained from the Brandaris 128 ultra-high-speed recordings during ultrasound (50 kPa peak negative pressure (PNP), 1.6 MHz). Scale bar is 1 µm and applies to all images.

Based on the physicochemical characterization described above, indirect DSPC-based microbubbles with 12 mol% cholesterol were chosen for acoustical characterization because they had the most homogeneous ligand and lipid phase distribution. They were compared to the direct and indirect DSPC-based microbubbles without cholesterol, and for each type of microbubble, data were acquired from at least two separate batches. Figure 5 shows a typical example of a 3D confocal acquisition before and after ultrasound with the corresponding *R-t* curve obtained from the ultra-high-speed recording at 50 kPa PNP for a direct DSPC (top row), indirect DSPC (middle row), and indirect DSPC-cholesterol (bottom row) microbubble. The coating of direct and indirect DSPC microbubbles was phase-separated into dark LC domains with a bright interdomain region, while the coating of indirect DSPC-cholesterol microbubbles was in one homogeneous lipid phase. This was in line with the results obtained by 4Pi confocal microscopy. The direct DSPC microbubble shown in Figure 5 had one bright spot present in the coating before and after the ultrasound, which was classified as a buckle. The coating of the indirect DSPC microbubble in Figure 5 had one large and several smaller LC phase domains. For the indirect DSPC-cholesterol microbubble in Figure 5, the maximum intensity projection of the confocal *z*-stacks resulted in more brightness near the edge of the microbubble than in the center. However, when looking at the separate *z*-slices, the fluorescent signal was homogeneous over the microbubble coating (Supplemental Figure S3).

**Figure 6:**
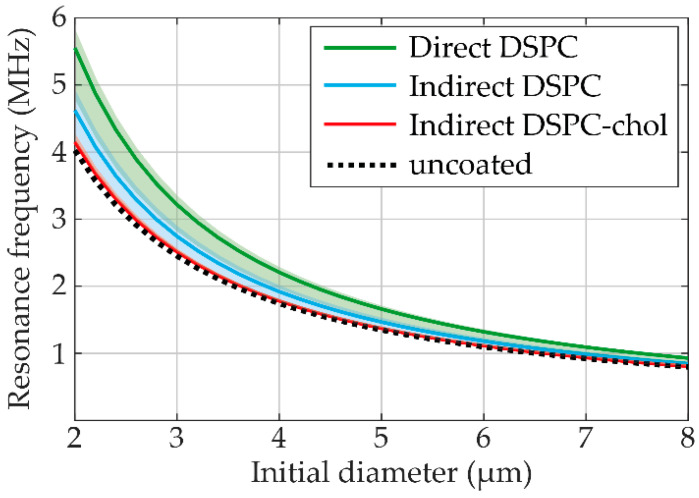
Resonance frequency (MHz) per initial diameter (µm) at 50 kPa of direct DSPC (green), indirect DSPC (blue), and indirect DSPC-cholesterol (red, 12 mol%) microbubbles. The dotted line represents the resonance frequency of uncoated microbubbles. The shaded areas indicate the range of individual microbubble resonance frequencies obtained by fitting at each *f_T_*.

Oscillation amplitudes at frequencies between 1 and 4 MHz and acoustic pressure of 50 kPa were obtained per microbubble from the *R-t* curves and fitted to the harmonic oscillator model at each *f_T_* (examples at 1.2, 1.6. and 2.0 MHz shown in Supplemental Figure S4). Resonance frequencies resulting from the fit to the harmonic oscillator model are presented in Figure 6, with the obtained shell elasticity and viscosity parameters listed in Table 1. The shell elasticity of direct DSPC microbubbles was the highest, while the shell elasticity of indirect DSPC-cholesterol microbubbles was close to that of an uncoated microbubble. The shell viscosity parameter is related to the damping of the oscillation and was lowest for the direct DSPC microbubbles, which had the highest oscillation amplitudes.

**Figure 7:**
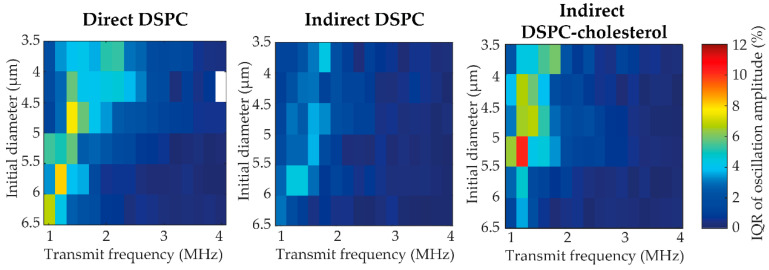
Variability in acoustic response represented as the IQR of the oscillation amplitude at 50 kPa of direct DSPC, indirect DSPC, and indirect DSPC-cholesterol (12 mol%) microbubbles of different sizes (3.5–6.5 µm) at different transmit frequencies (1–4 MHz). All bins are based on *N* > 3; bins with *N* < 3 are blank.

Figure 7 illustrates the variability in acoustical response within the three types of microbubbles. The variability was quantified as the interquartile range (IQR) of the oscillation amplitude from different microbubbles of the same size at the same transmit frequency (*N* > 3 per bin). The maximum and median IQR values for each type of microbubble are listed in Table 1. Indirect DSPC-cholesterol microbubbles had the highest maximum IQR, while direct DSPC microbubbles had the highest median IQR. Overall, indirect DSPC microbubbles exhibited the lowest variability in acoustical response.

**Figure 8:**
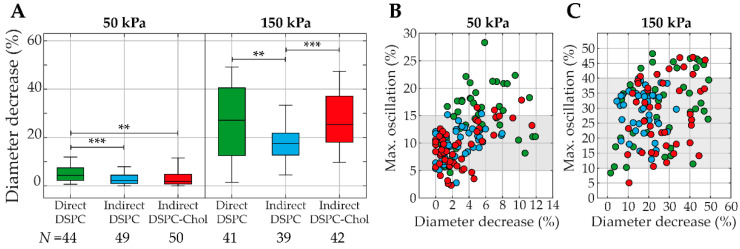
(**A**) Diameter decrease (%) for direct DSPC (green), indirect DSPC (blue), and indirect DSPC-cholesterol (red, 12 mol% cholesterol) microbubbles at 50 kPa (left panel) and 150 kPa (right panel). Boxplots represent the median and IQR. ** *p* < 0.01. *** *p* < 0.001. (**B**,**C**) Maximum oscillation (%) as a function of diameter decrease (%) for direct DSPC (green), indirect DSPC (blue), and DSPC-cholesterol (red, 12 mol% cholesterol) microbubbles at 50 kPa (**B**) and 150 kPa (**C**). Gray areas indicate the range of maximum oscillation values reached by all three types of microbubbles.

Figure 8 shows the deflation of the microbubble, quantified as the diameter decrease relative to the initial diameter, for direct DSPC, indirect DSPC, and indirect DSPC-cholesterol microbubbles. At 50 kPa, direct DSPC microbubbles deflated significantly more than the indirect DSPC and DSPC-cholesterol microbubbles, while no statistically significant difference in deflation was found between the indirect DSPC and DSPC-cholesterol microbubbles. However, at 50 kPa, the direct DSPC microbubbles had higher oscillation amplitudes than the other two groups. When comparing the deflation of microbubbles with similar oscillation amplitudes, marked as a gray area in Figure 8B, no statistically significant differences were found. Therefore, the statistical differences found at 50 kPa can be explained by a difference in oscillation amplitude, not acoustical stability. At 150 kPa, all types of microbubbles deflated significantly more than at 50 kPa. Furthermore, the indirect DSPC microbubbles deflated significantly less than both other groups, also when comparing only microbubbles with similar oscillation amplitudes (Figure 8C). No statistically significant difference in deflation was found between direct DSPC and indirect DSPC-cholesterol microbubbles.

**Figure 9:**
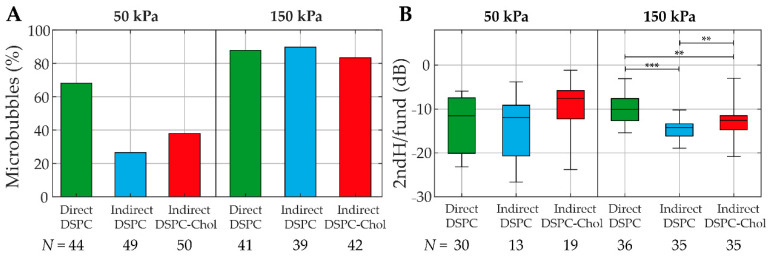
(**A**) Percentage of direct DSPC (green), indirect DSPC (blue), and indirect DSPC-cholesterol (red, 12 mol% cholesterol) microbubbles with a second harmonic response at 50 kPa (left panel) and 150 kPa (right panel). (**B**) Second harmonic amplitude normalized to the fundamental (dB) of direct DSPC (green), indirect DSPC (blue), and indirect DSPC-cholesterol (red, 12 mol% cholesterol) microbubbles at 50 kPa (left panel) and 150 kPa (right panel). Boxplots represent the median and interquartile range (IQR), with whiskers ranging from maximum to minimum. ** *p* < 0.01. *** *p* < 0.001.

The nonlinear behavior was studied by looking at the acoustic response at the subharmonic and second harmonic frequencies at 50 and 150 kPa. At subharmonic frequencies, all types of microbubbles had a low response rate, and no statistical differences were found between the groups (Supplemental Figure S5). The percentages of microbubbles with a response at the second harmonic frequency are presented in Figure 9A. At 50 kPa, the direct DSPC microbubbles exhibited the highest number of second harmonic responses (68%), while this number was considerably lower for the indirect DSPC (26%) and the indirect DSPC-cholesterol (38%) microbubbles. At 150 kPa, all three types had similar percentages of microbubbles with a second harmonic response, and all occurrences were higher than those at 50 kPa. The second harmonic amplitudes were similar for all microbubble types at 50 kPa (Figure 9B). At 150 kPa, however, the direct DSPC microbubbles had significantly higher second harmonic amplitudes than both other microbubble types. Additionally, the indirect DSPC-cholesterol microbubbles had a significantly higher second harmonic amplitude than the indirect DSPC microbubbles.

**Figure 10:**
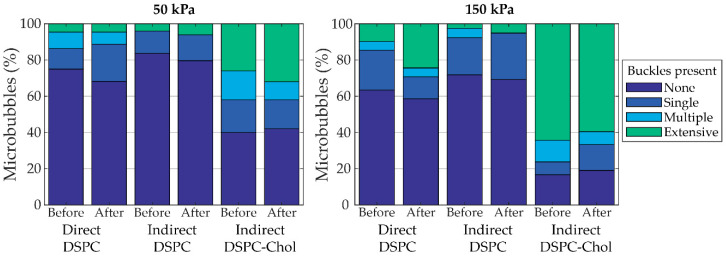
Percentage of microbubbles with buckles (none, single, multiple, or extensive) before and after insonification at 50 kPa (**left panel**) and 150 kPa (**right panel**). Indirect DSPC-cholesterol microbubbles contained 12 mol% cholesterol.

**Figure 11:**
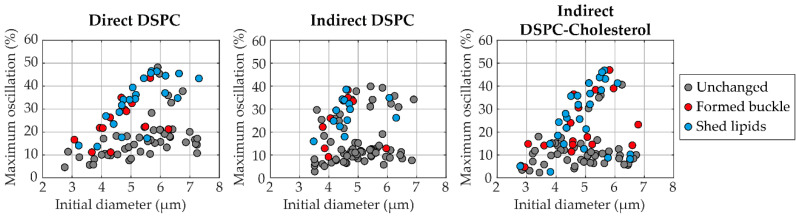
Maximum oscillation amplitude (%) of single direct DSPC (**left**, *N* = 85), indirect DSPC (**middle**, *N* = 88), and indirect DSPC-cholesterol (**right**, 12 mol% cholesterol, *N* = 92) microbubbles as a function of the initial microbubble diameter (µm). Microbubbles imaged by confocal microscopy directly before and after insonification (1–4 MHz, 50 or 150 kPa) were compared and scored as unchanged (gray), formed a buckle (red) or shed lipid material (blue).

Confocal *z*-stacks of each microbubble were manually scored for the presence of buckles (none, single, multiple, or extensive with examples provided in Supplemental Figure S2) before and after ultrasound insonification (Figure 10). Indirect DSPC microbubbles (*N* = 49 at 50 kPa; *N* = 39 at 150 kPa) had the lowest occurrence of buckles both before and after ultrasound insonification, which was comparable to that of the direct DSPC microbubbles (*N* = 44 at 50 kPa; *N* = 41 at 150 kPa). Indirect DSPC-cholesterol microbubbles (*N* = 50 at 50 kPa; *N* = 42 at 150 kPa) had a notably higher occurrence of buckles than both other groups at both 50 and 150 kPa. Further analysis did not reveal a direct correlation between the oscillation amplitude and the presence of buckles in the shell before ultrasound insonification (Supplemental Figure S6). The maximum oscillation amplitude was compared between microbubbles without buckles, with a single buckle, with multiple buckles, or with extensive buckles in the coating before ultrasound insonification. For all types of microbubbles, at 50 and 150 kPa, no statistically significant differences in oscillation amplitude were found between the groups. Next, the correlation between the change in microbubble coating upon ultrasound insonification and the maximum oscillation amplitude was evaluated, as shown in Figure 11. The median excursion amplitude of microbubbles that experienced a change, either by forming a buckle or by shedding lipids from the coating, was significantly larger (*p* < 0.001) than the excursion amplitude of unchanged microbubbles for all microbubble types. For direct DSPC microbubbles, the difference between changed and unchanged coatings was the most explicit, with a threshold amplitude of approximately 20% above which most microbubbles were changed after ultrasound insonification. For indirect DSPC microbubbles, the threshold amplitude was similar, albeit less pronounced. The indirect DSPC-cholesterol microbubbles also exhibited the formation of buckles and shedding of lipid material in microbubbles oscillating with amplitudes <20%.

**Figure 12:**
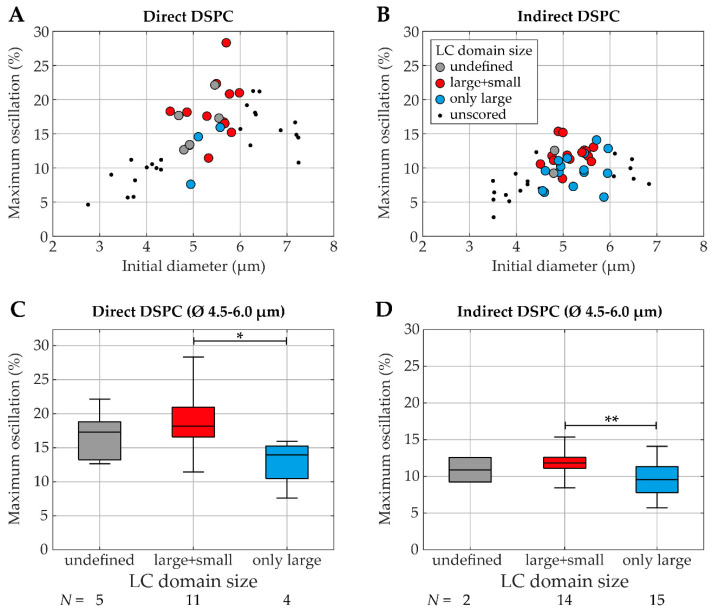
Maximum oscillation amplitude (%) at 50 kPa (over 1–4 MHz) as a function of initial diameter (µm) (**A**,**B**) for single direct DSPC (left) and indirect DSPC (right) microbubbles of 4.5–6.0 µm in diameter with LC domain size score as undefined (gray), large and small (red) or only large LC domains (blue), and as boxplot (**C**,**D**) representing the median and IQR with whiskers ranging from maximum to minimum. In A and B, the unscored microbubbles outside the 4.5–6.0 µm range are shown as black dots. * *p* < 0.05. ** *p* < 0.01.

Finally, the correlation between LC domain size and oscillation amplitude was investigated for a limited size range of microbubbles, ruling out size-dependent differences in oscillation (Figure 12). Since the indirect DSPC-cholesterol microbubbles were lacking LC domains, they could not be scored for their LC domain size. Unscored microbubbles are shown as black dots in Figure 12. For the direct and indirect DSPC microbubbles of 4.5–6.0 µm (initial diameter), the lipid phase distribution was scored as “only large LC domains”, “large and small LC domains”, or “undefined” (Supplemental Figure S7). Both the direct (*N* = 11) and indirect (*N* = 14) DSPC microbubbles with large and small LC domains had a significantly higher oscillation amplitude than those with only large LC domains (direct: *N* = 4, indirect: *N* = 15).

## 4. Discussion

The results of this study showed that cholesterol significantly affected the ligand and lipid phase distribution in DSPC-based phospholipid-coated microbubbles made by the indirect method. The lipid handling prior to microbubble production also affected the ligand distribution, as shown previously [16]. Both the addition of cholesterol and the lipid handling prior to microbubble production were shown to influence the acoustic behavior of the microbubbles, as reflected in the apparent elasticity and viscosity values and resonance frequencies. Finally, the variability in acoustic response was enhanced for the microbubbles without lipid phase separation in the coating, namely the indirect DSPC-based microbubbles with 12 mol% cholesterol.

### 4.1. Physicochemical Characterization

The first part of this study revolved around the production and physicochemical characterization of DSPC-based microbubbles with cholesterol. Results indicated that the mean size of the microbubbles decreased with increasing concentrations of cholesterol. In contrast, Kaur et al. found that microbubbles with DSPC and cholesterol (1:1 molar ratio) were not significantly different in size from microbubbles with DSPC only [25]. However, those microbubbles were air-filled and did not contain any emulsifier such as PEG40-stearate or DSPE-PEG2000 like the microbubbles investigated in the present study. In our study, the span value increased with increasing concentrations of cholesterol, indicating that microbubbles with cholesterol were more polydisperse than those without cholesterol. Furthermore, the variability in polydispersity was larger between batches of microbubbles with cholesterol than those without cholesterol.

The addition of cholesterol to the indirect DSPC-based microbubble coating affected both the ligand and the lipid phase distribution. Indirect DSPC microbubbles without cholesterol had a mostly homogeneous ligand distribution as shown by fluorescence microscopy imaging, which is in agreement with results from Langeveld et al. [16]. However, all types of microbubbles with cholesterol had significantly more heterogeneous and variable ligand distribution than those without cholesterol. While the ligand distribution of microbubbles with 12 mol% cholesterol was the most homogeneous and comparable to that of the indirect DSPC microbubbles without cholesterol, indirect DSPC microbubbles with 14 mol% cholesterol had a more heterogeneous ligand distribution. The increased number of buckles in the coating is likely the reason for this increase in heterogeneity.

The indirect DSPC microbubbles without cholesterol had a lipid phase distribution similar to previous reports, with dark LC domains and a bright interdomain LE region [14,16]. All types of microbubbles with cholesterol had a significantly smaller LC phase area than those without cholesterol, indicating that cholesterol molecules modified the lateral molecular packing of the microbubble coating. The impact of cholesterol on the lipid phase distribution was most evident in microbubbles with 12 mol% cholesterol, where all components appeared to be miscible and in a single homogeneous phase. With a higher concentration of cholesterol, specifically 14 mol%, the quantified LC phase area was larger than in microbubbles with 12 mol% cholesterol. A previously reported analysis of the lipid phase behavior in binary monolayers of DPPC or DSPC with cholesterol suggested a three-state phase model [23], where cholesterol either reduced or increased the lateral molecular packing. According to that study, the lateral molecular packing of a lipid monolayer is expected to decrease with low concentrations of cholesterol and increase with higher concentrations of cholesterol. This is in agreement with our results of the lateral molecular packing, quantified here as LC phase area, decreasing up to 12 mol% and then increasing at 14 mol% cholesterol. Other work focused on lipid phase behavior in monolayers includes atomic force microscopy images of monolayers with DPPC and 33 mol% cholesterol, showing a homogeneous phase distribution [22]. While we found microbubbles with 32 mol% cholesterol to be highly unstable, those with 12 mol% had a homogeneous phase distribution. This suggests that the phase behavior of phospholipids in a monolayer cannot be directly translated to the phase behavior of phospholipids in a microbubble coating, which is supported by a direct comparison of lipid phase behavior in monolayers and microbubble coatings with the same ternary mixture of DPPC or DSPC with DSPE-PEG2000 and PEG40-stearate [16].

Interestingly, cholesterol (10–50 mol%) has been used for many years to stabilize liposomes with DPPC or DSPC by increasing the lateral molecular packing [34], emphasizing the difference in lamellar structures, i.e., bilayers, of a liposome compared to the phospholipid monolayer coating of a microbubble. DSPC forms lamellar structures when suspended in water at room temperature [35]. However, during microbubble production by probe sonication, the lamellar structures are disrupted, and the molecules self-assemble as a monolayer of phospholipids at the gas–liquid interface [36]. In a model membrane system with monolayer-bilayer junctions, cholesterol was shown to be involved in lipid-driven budding of the membrane, with higher concentrations of cholesterol resulting in increased budding [37]. These findings are in agreement with the increased budding and formation of buckles we found in microbubbles with higher concentrations of cholesterol in the coating. In this context, budding refers to the formation of lipid bilayer-coated vesicles, while buckle formation refers to bilayers that are still attached to the lipid monolayer coating of the microbubble.

The present study includes a normalization factor in the analysis of the 4Pi microscopy data to compensate for a difference in fluorescence intensity between the middle and the top or bottom of the microbubbles. The normalization factor did not affect the proper quantification of the LC phase area in microbubbles without cholesterol. Since the difference in fluorescence signal between LC and LE phase in those microbubbles was much larger than the difference in signal between the middle and top or bottom of the microbubble, the LC phase area could easily be quantified in microbubbles with clear separation of the lipids into LC and LE phase. The imaging artifact only became evident during the analysis of microbubbles with a homogeneous lipid phase distribution, i.e., containing cholesterol. All experiments in this study were performed at a room temperature of 19–21 °C. Since the 4Pi confocal microscope operates at a limited range of temperature, this practice facilitated comparison of the data obtained from the 4Pi confocal microscopy and the acoustic characterization with the combined confocal and Brandaris 128 system and was in accordance with previous microscopy studies on lipid and ligand distribution in microbubble coatings [14,16]. Slight fluctuations in the temperature of the sample due to, for instance, the light or ultrasound are not expected to affect the lipid phase distribution, since the transition temperature for DSPC is 55 °C [38]. Furthermore, it was previously reported that in lipid bilayers of DPPC and cholesterol (10 or 20 mol%), the lipid phase distribution was only affected by temperatures above 40 °C [39]. Processing of lipid films in the sonicator bath and with the probe sonicator at power 3 did not affect the temperature of the samples.

### 4.2. Acoustical Characterization

Microbubble spectroscopy was performed on direct DSPC, indirect DSPC, and indirect DSPC-cholesterol (12 mol%) microbubbles to characterize their acoustic behavior. The shell parameters found here can be directly compared to a previous study by van Rooij et al. [11], which used a similar method and included the same direct DSPC microbubbles as the current study. The shell elasticity found in the present study (0.14 (0.12–0.15) N/m) (median (IQR)) was slightly lower and the shell viscosity (0.43 (0.38–0.61) × 10^−8^ kg/s) slightly higher than previously published (0.26 ± 0.13 N/m, mean ± SD; 1.0 (0.7) × 10^−8^ kg/s, median (IQR)), however, still within the error margins. The indirect DSPC microbubbles had a shell elasticity approaching that of an uncoated microbubble, similar to the DPPC-based microbubbles studied by van Rooij et al. [11]. As the shell elasticity was lower, the resonance frequency was also lower (Equation (2)). Both the indirect DSPC and the indirect DSPC-cholesterol microbubbles had a higher shell viscosity than the direct DSPC microbubbles. This was reflected in the oscillation amplitudes at 50 kPa, which were higher for the direct DSPC microbubbles than for the other groups, indicating lower damping and, therefore, lower viscosity (Equation (1)).

The influence of lipid phase distribution and lipid handling on the variability in acoustic response was assessed by comparing microbubbles with lipid phase separation, i.e., indirect DSPC, to those without lipid phase separation, i.e., indirect DSPC-cholesterol (12 mol%), and to those made with a different way of lipid handling prior to microbubble production, i.e., direct DSPC. While indirect DSPC-cholesterol microbubbles had the highest maximum variability in response, the median variability was highest for the direct DSPC microbubbles. These results suggest that lipid handling prior to microbubble production can reduce the variability in response and that although the maximum variability was highest in the indirect DSPC-cholesterol microbubbles, the difference in lipid phase separation did not affect the variability in acoustic response overall. Due to their more uniform response, the indirect DSPC microbubbles would be the most suitable candidate for drug delivery applications. Two maxima can be observed in the variability in the response ofdirect and indirect DSPC microbubbles to ultrasound insonification. While this may be explained as a size-dependent effect, it is not a distinct trend and perhaps more likely due to the limited sample size. Apart from differences between the microbubble types, all microbubbles exhibited the highest variability in response at the resonance frequency. Thus, to insonify microbubbles at a frequency other than their resonance frequency could be a new strategy to achieve a more uniform response to ultrasound, although monodisperse microbubbles are needed for this strategy to yield a uniform and predictive response of a bulk of microbubbles.

Acoustical stability was studied using the decrease in diameter after ultrasound, i.e., deflation. For this analysis, the mean microbubble diameter of 128 frames without ultrasound was regarded as the initial diameter. Since the final diameter was determined based on the last recording of each microbubble, i.e., the recording of ultrasound insonification at 4 MHz, only the last 10 frames were used to calculate the mean microbubble diameter. The microbubble size in these last frames was stable, and the difference in sample size is therefore not expected to influence the results. At 50 kPa, statistical differences in deflation could be explained by differences in the oscillation amplitude. At 150 kPa, however, the indirect DSPC microbubbles were significantly more stable than the direct DSPC and indirect DSPC-cholesterol microbubbles. Since no statistical differences were found between the direct DSPC and indirect DSPC-cholesterol microbubbles, the difference in acoustical stability was not caused by a difference in coating microstructure, which is in accordance with previous studies [40]. As the composition of direct and indirect DSPC microbubbles was exactly the same, the difference in acoustical stability must have been caused by the difference in lipid handling prior to microbubble production, which is known to alter the ligand distribution, synonymous to the distribution of DSPE-PEG2000 [16].

The nonlinear behavior of microbubbles is imperative for successful contrast-enhanced ultrasound imaging and ultrasound molecular imaging. At 50 kPa, the direct DSPC microbubbles had a more frequent second harmonic response than the other types of microbubbles. At 150 kPa, the majority of all types of microbubbles had a second harmonic response. The differences in variability in the acoustic response between the types of microbubbles, as presented in Figure 7, are also reflected in the range of the second harmonic response at 150 kPa, presented in Figure 9B. Indirect DSPC microbubbles had the lowest variability in second harmonic amplitude at 150 kPa, while the response at 50 kPa was comparable to the other types. This could be due to the low amplitudes of the second harmonic response at 50 kPa, which translates to a larger experimental error. The percentage of direct DSPC microbubbles with a nonlinear response, subharmonic or second harmonic, at 50 kPa was lower than published before [11]. This may be explained by the different pulse lengths (8 cycle- instead of 10-cycle pulse) or the fact that as a lower amount of light reaches the Brandaris 128 camera in the current imaging system, the noise level is slightly higher. More experiments focused on nonlinear behavior are needed for a comprehensive assessment of indirect DSPC microbubbles for ultrasound molecular imaging. However, this lies outside the scope of the present study.

### 4.3. Lipid Phase Distribution and Acoustical Behavior

The homogeneous lipid phase distribution in indirect DSPC-cholesterol microbubbles found by 4Pi confocal microscopy was confirmed with confocal microscopy of the microbubbles also analyzed acoustically. Besides the homogeneous lipid phase distribution, indirect DSPC-cholesterol microbubbles had buckles in their coating before insonification more frequently and more extensively than microbubbles without cholesterol, demonstrated by 4Pi confocal microscopy as well. The DSPC-cholesterol microbubbles insonified at 150 kPa had more buckles than those insonified at 50 kPa, underlining the heterogeneity between different indirect DSPC-cholesterol microbubbles from the same batch. The variable buckle incidence may be explained by the low stability of the DSPC-cholesterol coating. Due to the low amount of LC phase area in their coating, indirect DSPC-cholesterol microbubbles are expected to dissolve at a faster rate than those without cholesterol [41]. Different collapse and shedding mechanisms, such as budding, folding, and buckling, have been proposed to explain how the phospholipid monolayer around the gas core responds to the spontaneous dissolution of the microbubble [42].

Next, the correlation between maximum oscillation amplitude and change in the microbubble coating was investigated. After combining the data from microbubbles insonified at 50 and 150 kPa, the oscillation amplitude of microbubbles that experienced change due to ultrasound insonification was found to be significantly higher than that of unchanged microbubbles for all microbubble types, with a threshold amplitude of approximately 20%. Other studies investigating the lipid coating behavior in microbubbles during ultrasound insonification found comparable results, namely a threshold oscillation amplitude of 30% [43,44]. The difference in threshold amplitude may be explained by the microbubble formulation as microbubbles in other studies were coated with DPPC [43] or DSPC [44] and DSPE-PEG2000, without PEG40-stearate. Another explanation could be a difference in the production method, as the microbubbles for the present study were all made by probe sonication, in contrast to the vial shaker method used for previous studies.

This study is the first to record both the lipid phase distribution and acoustic response in single microbubbles with the combined confocal microscope and Brandaris 128 camera system. Whereas no correlation could be confirmed between the oscillation amplitude and the amount of buckles present before insonification, the LC domain size did correlate with the oscillation amplitude. Microbubbles with small-sized LC domains had higher oscillation amplitudes, which is in accordance with previous reports on phospholipid-coated microbubbles with different sized LC domains, where those with smaller LC domains had a lower resistance to deformation [45]. By contrast, another study found no significant differences in the behavior or stability of microbubbles during ultrasound insonification when they related the lateral molecular packing to the acoustic behavior of microbubbles in different formulations, even though the method of production did affect the lipid packing significantly [46]. The reason for this could be the quantification of the lateral molecular packing, as this was done by calculating the generalized polarization value for single microbubbles, which does not account for lipid phase separation or microstructures in the coating. Before the DSPC-based microbubbles with and without cholesterol studied here can be used for in vivo applications, several differences between the in vitro setting and the in vivo situation need to be considered. Besides the temperature, these differences also include the blood flow, blood viscosity, and soft boundaries affecting the microbubbles’ acoustic behavior. Furthermore, the targeting strategy must be adapted to avoid an immune response to streptavidin, a foreign protein [47].

### 4.4. Implications of the Study

The addition of cholesterol to the indirect DSPC-based microbubble coating increased the variability in ligand distribution, acoustic response, polydispersity, and buckle formation. These effects can be explained by the altered lipid phase distribution as described above and imply that the indirect DSPC-cholesterol microbubbles are less stable than those without cholesterol. Because the indirect DSPC-cholesterol microbubbles had heterogeneities in the form of buckles, they could not be regarded as microbubbles with a uniform lipid distribution when comparing their acoustic behavior to that of the microbubbles with heterogeneous lipid phase distribution, i.e., the direct and indirect DSPC microbubbles. Thus, reduced stability of the microbubble coating is expected when the components are all miscible and in the same LE phase, which will increase the heterogeneity of the microbubble population and thereby increase the variability of the acoustical response. Therefore, a different approach will be required to achieve a more uniform microbubble response to ultrasound, possibly by tailoring the LC phase domains, as our results suggest that differences in LC domain size can predict the relative oscillation amplitude.

## 5. Conclusions

We produced indirect DSPC-based microbubbles with 7, 10, 12, and 14 mol% cholesterol in the coating. Cholesterol reduced lipid phase separation in the microbubble coating, resulting in a single phase at 12 mol% where all components were miscible. Buckle formation was increased with the reduction of the LC phase area, suggesting increased spontaneous dissolution of the microbubbles. As the acoustic behavior of DSPC-based microbubbles made by the direct and indirect method was compared to that of indirect DSPC-based microbubbles with 12 mol% cholesterol, indirect DSPC microbubbles had the most uniform response to the ultrasound and were the most stable acoustically. They had a lower shell elasticity and higher shell viscosity than the direct DSPC microbubbles. The modified lateral molecular packing of indirect DSPC-cholesterol microbubbles resulted in the lowest shell elasticity and highest shell viscosity of all microbubble types. Direct DSPC microbubbles displayed more nonlinear acoustic behavior than the indirect DSPC and indirect DSPC-cholesterol microbubbles. Based on these results, we can conclude that both the lipid phase separation and lipid handling prior to microbubble production significantly affected the acoustic behavior of microbubbles. The indirect DSPC microbubbles had the most promising results with regard to stability and uniform ultrasound response. These are important traits for an ultrasound molecular imaging agent and for drug delivery applications, as the acoustic behavior of the microbubble must be predictable and controllable.

## Figures and Tables

**Table 1:** Microbubble (MB) spectroscopy results at 50 kPa.

MB Type	*N*	Shell Elasticity ^1^ (N/m)	Shell Viscosity ^1^ (×10^−8^ kg/s)	Max IQR of Oscillation Amplitude (%)	Median IQR of Oscillation Amplitude (%)
Direct DSPC	44	0.14 (0.12–0.15)	0.43 (0.38–0.61)	8.0	1.5
Indirect DSPC	49	0.03 (0.01–0.06)	0.99 (0.89–1.40)	4.5	0.6
DSPC-cholesterol ^2^	50	0.01 (0.01–0.02)	1.39 (0.97–1.55)	10.2	0.7

^1^ presented as median (IQR); ^2^ indirect DSPC-cholesterol microbubbles with 12 mol% cholesterol.

## Data Availability

The data presented in this study are available on request from the corresponding author. The data are not publicly available due to the complex nature and tailored analysis for the unique experimental set-ups used in this study.

## Supplementary Materials

The following are available online at https://www.mdpi.com/1999-4923/13/1/119/s1, Figure S1: Normalization factors for quantitative analysis of 4Pi confocal microscopy data, Figure S2: Examples of buckle score based on confocal microscopy, Figure S3: Confocal slices from *z*-stack acquisition of indirect DSPC-cholesterol (12 mol%) microbubble, Figure S4: Relative oscillation amplitude of single microbubbles as a function of the initial diameter, Figure S5: Percentage of microbubbles with subharmonic responses and amplitudes of subharmonic response, Figure S6: Shell buckles versus oscillation amplitude, Figure S7: examples of LC domain size score based on confocal microscopy. Table S1. Concentration of indirect DSPC microbubbles (MBs).
