# Supplementary material for: The Impact of Lipid Handling and Phase Distribution on the Acoustic Behavior of Microbubbles
Source: Pharmaceutics. 2021 Jan 19;13(1):119. doi: 10.3390/pharmaceutics13010119 (PMC7832861; doi:10.3390/pharmaceutics13010119)
Supplement: Supplementary file 1 [file pharmaceutics-13-00119-s001.pdf]

pharmaceutics

Supplemental Materials: The Impact of Lipid Handling and Phase Distribution on the Acoustic Behavior of Microbubbles

Simone A.G. Langeveld, Inés Beekers, Gonzalo Collado-Lara, Antonius F. W. van der Steen, Nico de Jong, Klazina Kooiman

Table S1. Concentration of indirect DSPC microbubbles (MBs).

Cholesterol (mol%)	*N*	Mean Concentration (× 10^8 MBs/mL)	SD (× 10^8) or Range (min – max × 10^8)
0	5	4.71	2.14
7	2	5.28	5.01–5.55
10	2	9.85	8.04–11.7
12	6	5.96	2.60
14	2	7.21	6.01–8.40

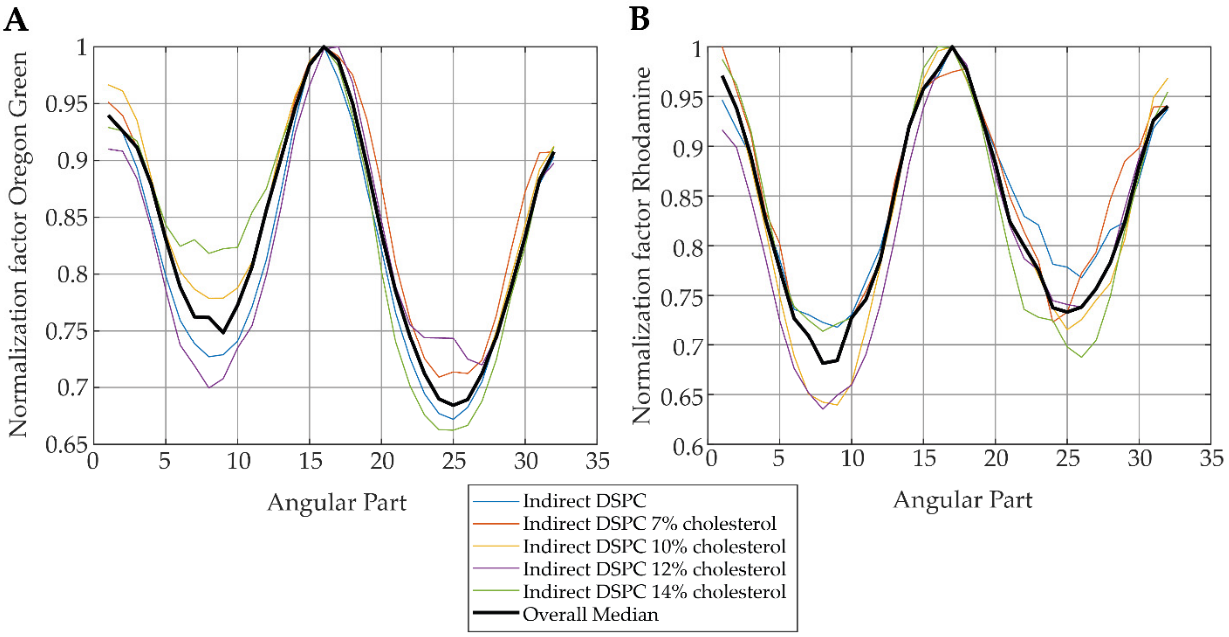

Figure S1. Normalization factors for quantitative analysis of 4Pi confocal microscopy data. A) Normalization factor for Oregon Green (green channel) per angular part. B) Normalization factor for Rhodamine (red channel) per angular part. The normalization factor is based on the difference in fluorescence intensity between angular parts in the center (part 5-10 and part 23-28) and the angular parts at the top and bottom of the microbubble (part 15-20, part 1-3, part 30-32). The fluorescence signal from the center of the microbubble is captured by both objectives, while the fluorescence signal from the top and bottom of the microbubble is captured by only one of the objectives. This is caused by attenuation of the signal and because the gas core of the microbubble has a different refraction index than the rest of the sample.

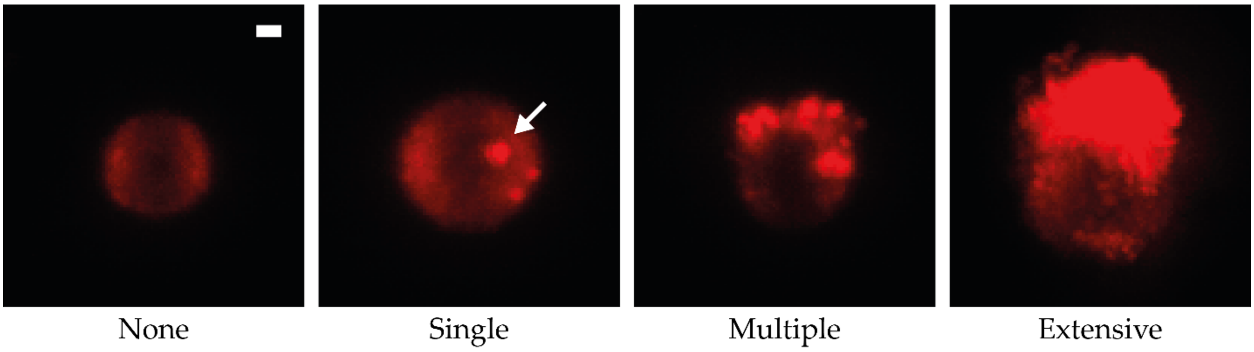

Figure S2. Confocal maximum intensity projections of *z*-stack from indirect DSPC-cholesterol (12 mol%) microbubbles with LE phase in red. Examples of buckle score: none, single, multiple, extensive. Bright spots with 1 µm diameter or larger were classified as a buckle. Scale bar represents 1 µm and applies to all images. In the single buckle example, the bright spot classified as buckle is indicated with a white arrow.

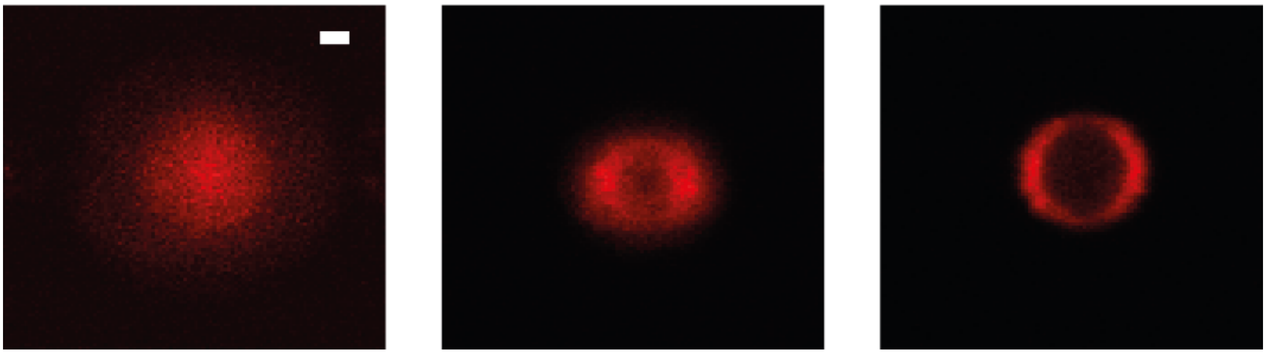

Figure S3. Confocal slices from *z*-stack acquisition of indirect DSPC-cholesterol (12 mol%) microbubble, presented as maximum intensity projection in Figure 5. Scale bar represents 1 µm and applies to all images. Positions of the slices (*z*-axis) are 0, 1.6 µm, 3.6 µm.

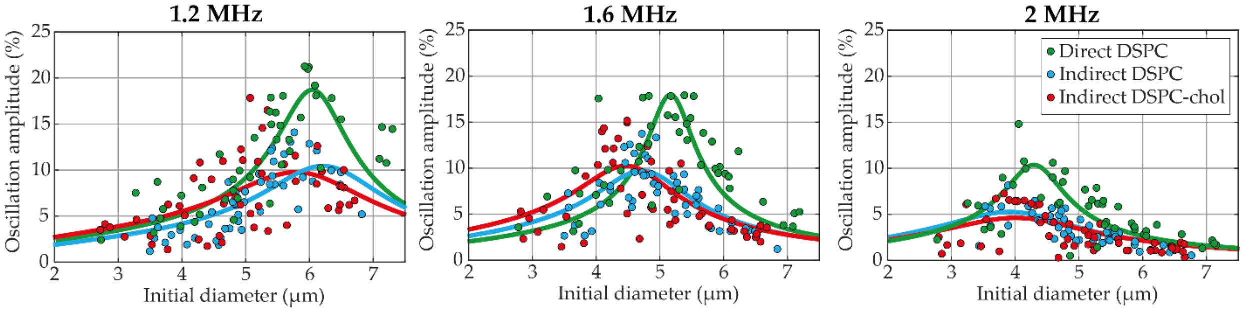

Figure S4. Relative oscillation amplitude (%) of single direct DSPC (green, *N* = 44), indirect DSPC (blue, *N* = 49), and indirect DSPC-cholesterol (red, *N* =50, 12 mol%) microbubbles as a function of the initial diameter when insonified at 50 kPa PNP with f_r = 1.2 MHz, 1.6 MHz, and 2 MHz. The lines in each plot represent the fit to the harmonic oscillator model for each microbubble type and at each f_r.

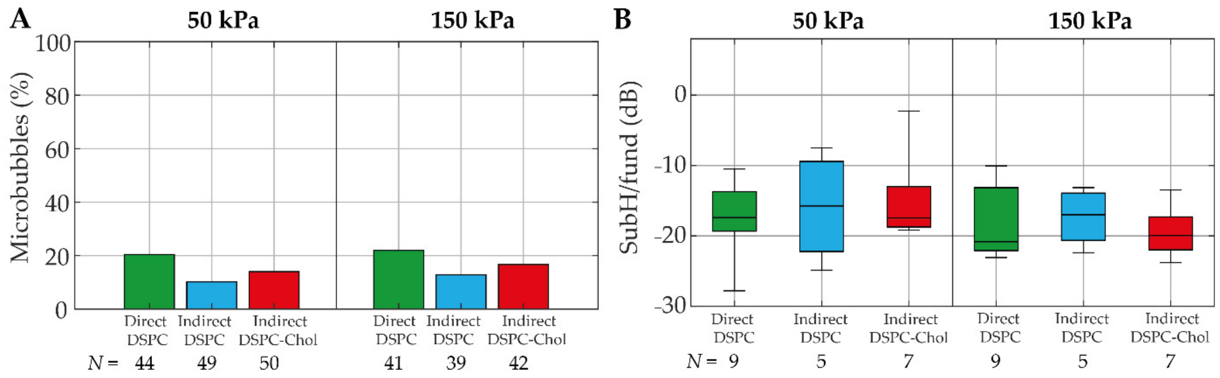

Figure S5. (**A**) Percentage of direct DSPC (green), indirect DSPC (blue), and indirect DSPC-cholesterol (red, 12 mol%) microbubbles with subharmonic response at 50 kPa (left panel) and 150 kPa (right panel). (**B**) Subharmonic amplitude (dB) normalized by the fundamental of direct DSPC (green), indirect DSPC (blue), and DSPC-cholesterol (green) microbubbles at 50 kPa (left panel) and 150 kPa (right panel). Boxplots represent the median and IQR.

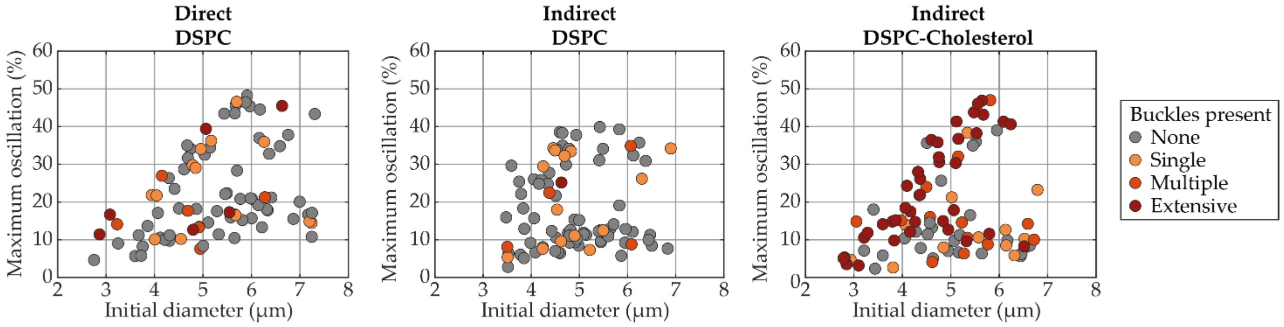

Figure S6. Shell buckles versus oscillation amplitude. Maximum oscillation amplitude (%) of single direct DSPC (left, $N = 85$), indirect DSPC (middle, $N = 88$), and indirect DSPC-cholesterol (right, $N = 92$, 12 mol%) microbubbles as a function of the initial diameter (µm). Microbubbles were scored for buckles present before insonification (1-4 MHz, 50 or 150 kPa) based on confocal *z*-stacks as none (grey), single (orange), multiple (bright red) or extensive (dark red).

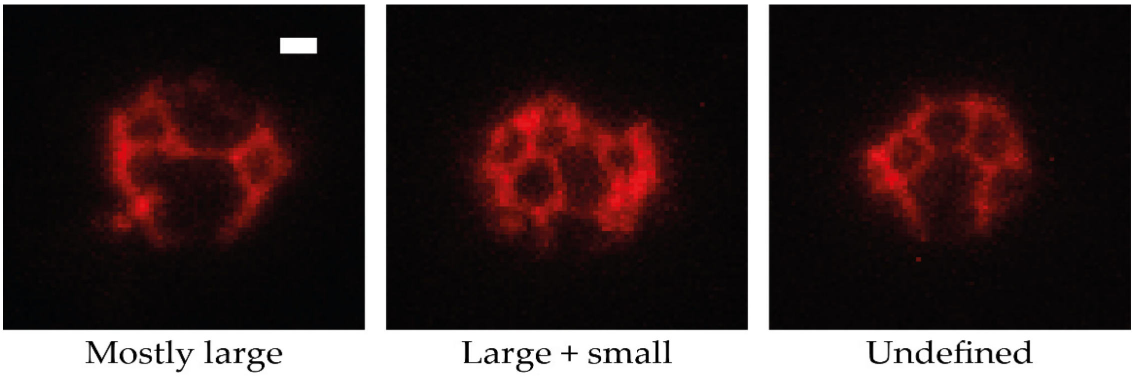

Figure S7. Confocal maximum intensity projections of z-stacks from indirect DSPC microbubbles with LE phase in red. Examples of lipid phase distribution scores: "only large LC domains", "large and small LC domains", or "undefined". Scale bar represents 1 µm and applies to all images.
